# Construction of a culture protocol for functional bile canaliculi formation to apply human iPS cell-derived hepatocytes for cholestasis evaluation

**DOI:** 10.1038/s41598-022-19469-x

**Published:** 2022-09-07

**Authors:** Shinichiro Horiuchi, Yukie Kuroda, Ryota Oyafuso, Yuji Komizu, Takashi Takaki, Kazuya Maeda, Seiichi Ishida

**Affiliations:** 1 Division of Pharmacology, National Institute of Health Sciences, Kawasaki, Japan; 2 Division of Applied Life Science, Graduate School of Engineering, Sojo University, Kumamoto, Japan; 3 Division of Electron Microscopy, Showa University, Tokyo, Japan; 4 Laboratory of Pharmaceutics, School of Pharmacy, Kitasato University, Tokyo, Japan; 5 Laboratory of Molecular Pharmacokinetics, Graduate School of Pharmaceutical Sciences, The University of Tokyo, Tokyo, Japan

**Keywords:** Drug development, Pharmacology, Drug safety

## Abstract

Cholestatic toxicity causes the failure of pharmaceutical agents during drug development and, thus, should be identified at an early stage of drug discovery and development. The formation of functional bile canaliculi in human hepatocytes is required for in vitro cholestasis toxicity tests conducted during the early stage of drug development. In this study, we investigated the culture conditions required for the formation of bile canaliculi using human-induced pluripotent stem cell-derived hepatocytes (hiPSC-Heps). When hiPSC-Heps were sandwich-cultured under the condition we established, extended bile canaliculi were formed on the whole well surfaces. Biliary efflux transporters were localized in the formed bile canaliculi structures which had junctional complexes. After the model substrates of the biliary efflux transporters were taken up into cells, their subsequent excretion into the bile canaliculi was observed and was found to be impeded by each inhibitor of the biliary efflux transporter. These findings suggest that bile canaliculi have transporter-specific bile excretion abilities. We will continue to study the application of this culture protocol to cell-based cholestasis assay system. As a result, the culture protocol could lead to a highly predictable, robust cell-based cholestasis assay system because it forms functional bile canaliculi reproducibly and efficiently.

## Introduction

The liver is the major organ for the uptake, metabolism, and excretion of drugs and other xenobiotics; thus, it is vulnerable to drug toxicity. Drug-induced liver injury (DILI) is one of the primary reasons for the failure of pharmaceutical agents during drug development and was the most common cause of the withdrawal of medicinal products from the market between 1953 and 2013^1^. DILI is classified as a hepatocellular, cholestatic, or mixed injury based on the major underlying mechanism. Among the 1676 DILI cases that occurred in Japan between 1997 and 2006, 40% of the patients had either cholestatic or mixed cholestatic hepatic toxicity^2^. Cholestatic liver injury, including the mixed hepatocellular/cholestatic type, is the most severe case. According to a historical survey, the exacerbation risk in a DILI patient is increased by cholestasis, and the fatality rate of the case can reach 10–50%^3^. Many drugs that cause either cholestatic or mixed hepatocellular/cholestatic liver injury have been reported, some of which (e.g., troglitazone and bosentan) were withdrawn from the market. Therefore, an exact evaluation of the drugs that cause cholestasis is required in drug development.

Bile is excreted from hepatocytes into bile canaliculi and transported into the duodenum via the common bile duct. Cholestasis is a condition in which bile flow is obstructed at some point in these processes. There are many reported causes of drug-induced cholestasis^3–5^; inhibition of bile acid transporters is one of the main ones^6–9^. Cholestatic drugs disrupt the function of bile acid transporters by direct inhibition or indirect processes, including the regulation of transporter localization or expression^10^. For example, troglitazone competitively inhibits the canalicular efflux transporter, bile salt export pump (BSEP)^11^. Canalicular efflux transporters are located in the canalicular membranes of hepatocytes and mediate bile excretion from the hepatocyte cytoplasm into the bile canaliculi. Therefore, the inhibition of canalicular efflux transporters induces cholestatic hepatocyte damage through the intracellular accumulation of bile acids.

Animal experiments have been conducted in the preclinical stage but were unable to significantly predict the liver injury associated with the BSEP interference observed in humans because of interspecies differences in bile acid composition, hepatobiliary transporter modulation or constitutive expression, and other mechanisms^12^. Membrane vesicle assays (MVAs) have been used to evaluate the risk of drug-induced bile acid transport inhibition and subsequent cholestatic DILI^13,14^. However, risk assessment of drug-induced cholestasis by MVA is insufficient because the effects of drug metabolism and interaction with other transporters cannot be evaluated. Therefore, a cell-based assay system that can accurately evaluate the effects of drugs on biliary efflux using human hepatocytes is needed to detect drugs that inhibit canalicular efflux transporters before clinical testing.

Sandwich-culture of hepatocytes has been utilized for bile canaliculi formation^15^. Sandwich-cultured hepatocytes regain polarity, allowing for the proper localization of basolateral and biliary transporters as well as bile canaliculi formation. However, the stable formation of extended bile canaliculi, which is required for a cholestasis toxicity test and biliary efflux evaluation, is difficult in human hepatocytes, even when applying sandwich culture. HEPATOPAC is one of the most used culture models for bile canaliculi formation and has been applied to the entrusted transporter assay in SOLVO Biotechnology. HEPATOPAC technology is a micropatterned co-culture system using primary hepatocytes and stromal cells, and the formation of extended bile canaliculi in human hepatocytes using that technology has been reported^16,17^. Hepatocytes in HEPATOPAC^®^ kits form “islands” when surrounded by supportive stromal cells in the culture vessel that was formed using a proprietary patterning method. This specialized architecture replicates the physiological microenvironment of the liver.

We have previously observed the extended bile canaliculi on a part of the well surface in a culture plate when human-induced pluripotent stem cell-derived hepatocytes (hiPSC-Heps) were cultured long-term. Thus, we attempted to form extended bile canaliculi on the whole well surface by introducing a sandwich culture into the long-term culture of hiPSC-Heps. Additionally, we evaluated the function of bile canaliculi formed in this protocol with the aim of applying hiPSC-Heps to cholestasis toxicity and biliary efflux evaluation.

## Results

### Establishment of conditions for the formation of extended bile canaliculi

**Figure 1:**
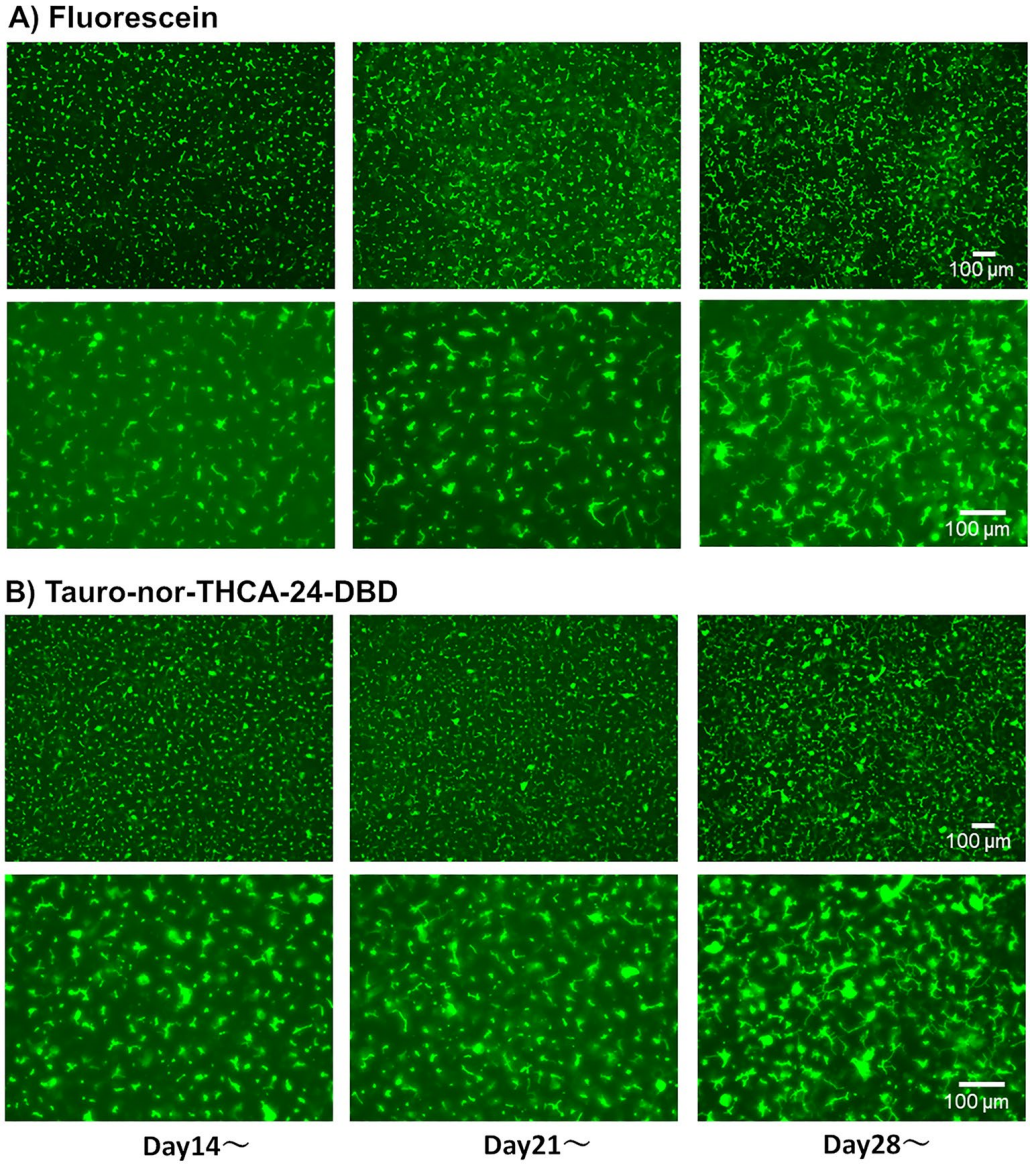
Accumulation of model fluorescein substrates into bile canaliculi when the culture conditions for the formation of extended bile canaliculi were examined. Human-induced pluripotent stem cell-derived hepatocytes were cultured in a long-term maintenance medium for 14 days, 21 days, and 28 days and then, they were sandwich-cultured in a maintenance medium for 9 days. Following the sandwich culture, the excretion of model fluorescein substrates (fluorescein diacetate and tauro-nor-THCA-24-DBD) into bile canaliculi was observed. Fluorescence images show (**A**) Fluorescein and (**B**) Tauro-nor-THCA-24-DBD, which were accumulated into bile canaliculi.

**Figure 2:**
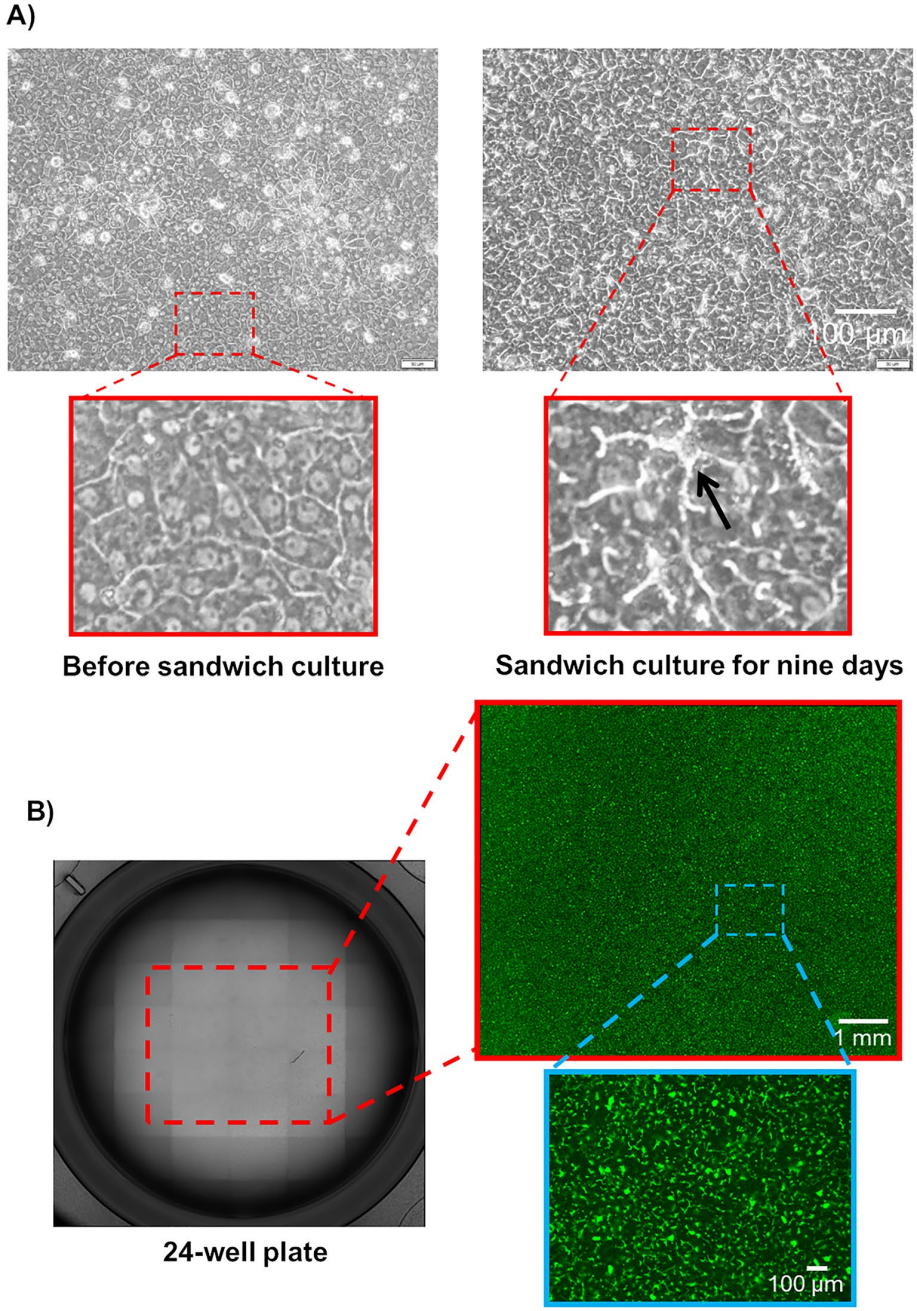
Bile canaliculi formed in suitable culture conditions. Human-induced pluripotent stem cell-derived hepatocytes were cultured in a long-term maintenance medium for 28 days and then, they were sandwich-cultured in a maintenance medium for 9 days. (**A**) The cell morphology that was observed using a phase-contrast microscope before and after the sandwich culture. The black arrow shows the extended bile canaliculi. After sandwich culture, the excretion of tauro-nor-THCA-24-DBD into a bile canaliculus was observed in a wide area within the well (**B**: Red dotted line). Fluorescence images show (**B**) Tauro-nor-THCA-24-DBD, which was accumulated into bile canaliculi.

**Figure 3:**
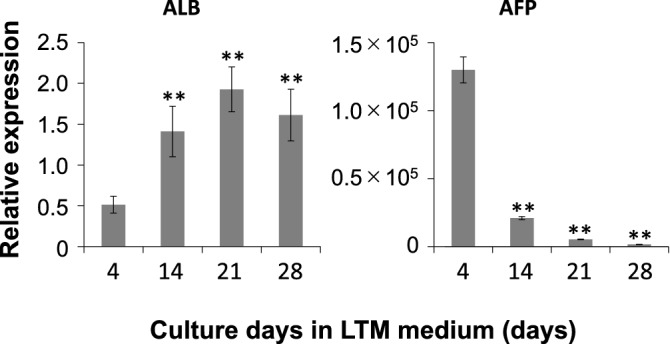
Expression of the genes which relate to the maturity of hepatocytes while cells were cultured in a long-term maintenance medium. The expression levels of *ALB* and *AFP* were measured using quantitative polymerase chain reaction over time during culture in a long-term maintenance medium for 28 days. The bar shows the relative expression levels of ALB or AFP. Pooled human liver RNA was used for the standard curve, and the expression level was set as one. The relative expression level was calculated using the equation of the line for the standard curve. Data are presented as means ± S.D. (n = 3). ** Shows that expression significantly increased compared with that on day 4 (P < 0.01).

The formation of extended bile canaliculi is difficult in human hepatocytes, and the vendor-recommended culture method originated only the dot-shaped bile canaliculi in iPSC-Hep (Fig. S1, top). However, when hiPSC-Heps were cultured in a Cellartis^®^ Enhanced hiPS-HEP Long-Term Maintenance Medium (LTM medium) for 30 days, extended bile canaliculi were observed on a part of the well surface in a collagen-coated culture plate (Fig. S1, bottom). Therefore, we screened the culture conditions for the formation of extended bile canaliculi on the whole well surface by improving the long-term culture conditions. hiPSC-Heps were sandwich-cultured following a 2D-culture for 21 days or longer for bile canaliculi formation (Fig. S2A). Before the sandwich culture, extended bile canaliculi were observed on a part of the well surface, but after the sandwich culture, extended bile canaliculi were observed on the whole well surface (Fig. S2B). When bile canaliculi were observed on the whole well surface, the biliary excretion to bile canaliculi was observed using fluorescein diacetate (FDA) and N-(24-[7-(4-*N*,*N*-dimethylaminosulfonyl-2,1,3-benzoxadiazole)]amino-3α,7α,12α-trihydroxy-27-nor-5β-cholestan-26-oyl)-2′-aminoethane-sulfonate (tauro-nor-THCA-24-DBD), which are fluorogenic substrates of biliary efflux transporters. FDA is taken up by cells and then hydrolyzed into fluorescein, which is transported into bile canaliculi by multidrug resistance protein 2 (MRP2)^18^. Tauro-nor-THCA-24-DBD, a synthetic fluorescent bile acid derivative, is transported into bile canaliculi via BSEP^19^. Fluorescein accumulation was not observed in part of the bile canaliculi formed on the well surface (Fig. S3). This may have happened because of insufficient expression of *MRP2*, which is a fluorescein efflux transporter. To solve this problem, the culture medium used for the sandwich culture was changed to a maintenance medium for iCell hepatocytes, which induced higher expression of *MRP2* than the LTM medium in our previous short-term culture (Fig. S4). As a result, extended bile canaliculi were observed on the whole well surface by fluorescence imaging using FDA and tauro-nor-THCA-24-DBD (Fig. S5). Next, the timing of the switch to a sandwich culture was examined to form the extended bile canaliculi efficiently (Fig. S6). When the sandwich culture was switched on day 14 or 21, the degree of extension was low, although bile canaliculi were formed on the whole well surface (Fig. 1). However, when the sandwich culture was switched on day 28, bile canaliculi were formed on the whole well surface and were well extended (Fig. 1). Under these conditions, extended bile canaliculi could be clearly observed with a phase-contrast microscope (Fig. 2A), and the accumulation of the fluorescent substrate was evenly observed across a wide area of the well (Fig. 2B). By culturing in an LTM medium for 28 days, the expression of *ALB*, which is an adult hepatic marker, was increased (threefold, compared to day 4) and that of *AFP*, which is a fetal hepatic marker, was decreased (1/80-fold, compared to day 4), suggesting that hiPSC-Heps were shifting towards adult hepatocytes (Fig. 3). In addition, the expression of CYP7A1, which relate to bile acid synthesis, was also increased by culturing in an LTM medium for 28 days (Fig. S7). The expression of the biliary efflux transporters, MRP2 and BSEP, and bile acid receptor, FXR, was at the same level as that in the human liver (relative expression = 1) and was maintained during 28 days of culture in an LTM medium (Fig. S7). Based on these results, the timing of switching to sandwich culture was determined to be day 28 in subsequent experiments.

### Function of biliary efflux transporters in the extended bile canaliculi

**Figure 4:**
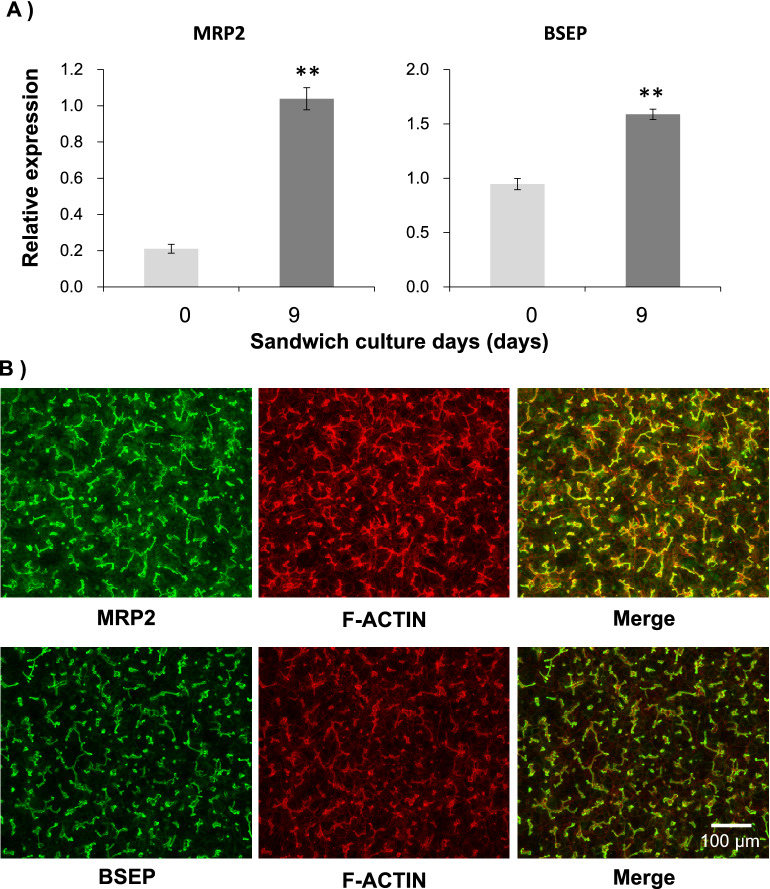
Gene expressions and the localization of biliary efflux transporters when the bile canaliculi were formed. Human-induced pluripotent stem cell-derived hepatocytes were cultured in a long-term maintenance medium for 28 days and then, they were sandwich-cultured in a maintenance medium for 9 days. (**A**) Expression levels of the biliary efflux transporters, multidrug resistance protein 2 (MRP2) and bile salt export pump (BSEP), were measured using quantitative polymerase chain reaction before and after sandwich culture. The bar shows the relative expression levels of *MRP2* or *BSEP*. Pooled RNA from human liver was used for the standard curve, and the expression level was set as one. The relative expression level was calculated using the equation of the line for the standard curve. Data are presented as means ± S.D. (n = 3). ** Indicates that the expression significantly increased compared with that prior to the sandwich culture (P < 0.01). (**B**) Immunostaining images of MRP2 and BSEP after sandwich culture. Actin filaments were stained with phalloidin as an index of bile canaliculi.

**Figure 5:**
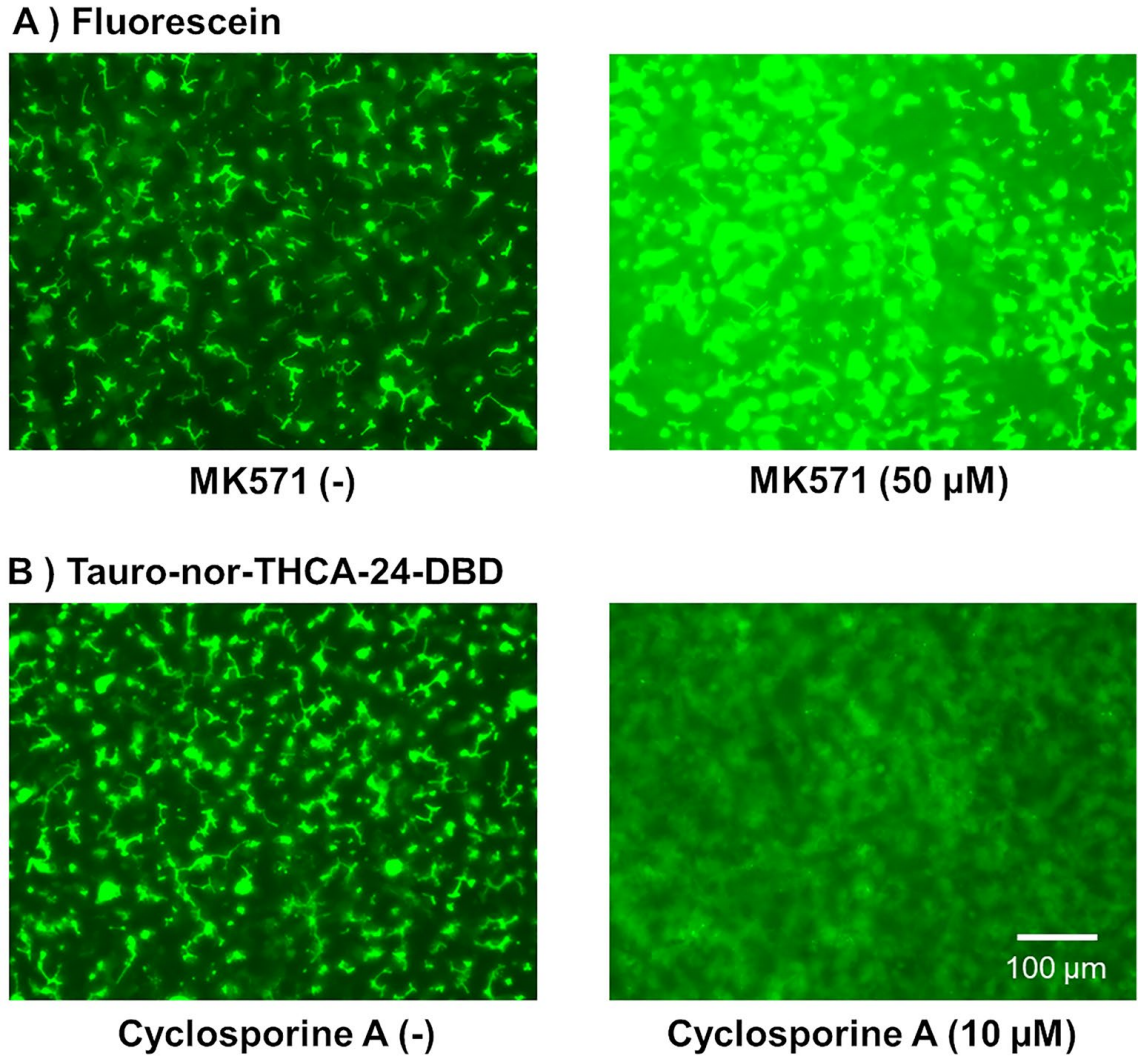
Effect of a biliary efflux transporter inhibitor on the excretion of the model substrate into bile canaliculi. Human-induced pluripotent stem cell-derived hepatocytes were cultured in a long-term maintenance medium for 28 days and then, they were sandwich-cultured in a maintenance medium for 9 days. After that, the effect of a biliary efflux transporter inhibitor on the excretion of the model substrate into bile canaliculi was examined. Fluorescence images show (**A**) Fluorescein or (**B**) Tauro-nor-THCA-24-DBD, when cells were treated with the biliary efflux transporter inhibitor (MK571 or cyclosporine A) or left untreated.

To confirm that biliary efflux transporters were functioning in the established culture condition, the expression of the biliary efflux transporters, *MRP2* and *BSEP*, and hepatic uptake transporters, *OATP1B1* and *NTCP*, was measured using quantitative PCR. The expression levels before and after sandwich culture are shown in Fig. 4A and Fig. S8. The expression levels of *MRP2*, *BSEP*, *OATP1B1* and *NTCP* increased for 9 days to levels similar to the human liver because of the sandwich culture. Since the localization of biliary efflux transporters to bile canaliculi is required for their function, immunostaining was performed to observe the localization of MRP2 and BSEP. Additionally, F-actin, which forms a structure that lines the bile canaliculi, was stained with phalloidin as a bile canaliculi structural marker. The stained images of MRP2 and BSEP overlapped with those of F-actin (Fig. 4B). This result suggests that MRP2 and BSEP are located in bile canaliculi. Biliary excretion was observed using FDA and tauro-nor-THCA-24-DBD, and both of the fluorescent substrates accumulated in the bile canaliculi (Fig. 5, left). Furthermore, the effects of the biliary efflux transporter inhibitor were examined to demonstrate that the fluorescein model substrate is excreted into the bile canaliculi via the biliary efflux transporter. K571, an MRP2 inhibitor^20^, inhibited fluorescein efflux into bile canaliculi and increased the amount of fluorescein remaining in the cells (Fig. 5A, right, Fig. S9A). The efflux of tauro-nor-THCA-24-DBD into bile canaliculi was inhibited by Cyclosporine A (CsA), a BSEP inhibitor^19^, the amount remaining in the cells increased, and accumulation in the bile canaliculi was not observed (Fig. 5B, right, Fig. S9B). These results confirmed the transporter-dependent canalicular efflux.

### Comparison of bile canaliculi formation between batches

hiPSC-Heps were independently cultured three times for bile canaliculi formation, and bile canaliculi formation was compared between batches. Similar images of fluorogenic substrate accumulation in bile canaliculi were found in all batches (Fig. S10). The reproducibility of the culture protocol for bile canaliculi formation was thus confirmed.

### Stability of canalicular efflux ability

**Figure 6:**
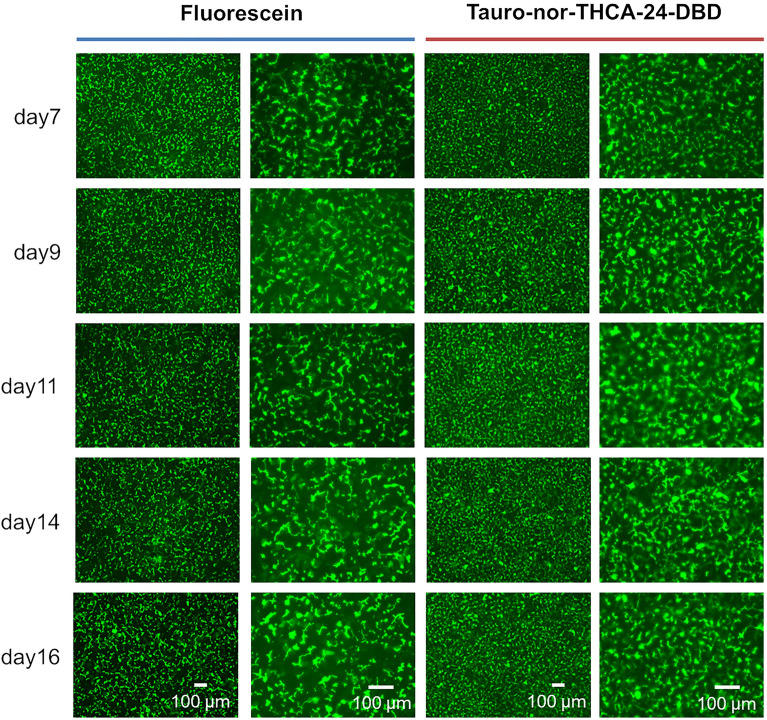
Accumulation of model fluorescein substrates into bile canaliculi when the stability of the canalicular efflux ability was examined. The term of sandwich culture in a maintenance medium following culture in an LTM medium for 28 days was examined to determine how long the assay is possible. Fluorescence images show fluorescein or tauro-nor-THCA-24-DBD, which accumulated in bile canaliculi when biliary efflux assay was performed on the 7th to 14th day of the sandwich culture.

Drug-induced cholestasis should be evaluated during the period when biliary efflux to bile canaliculi functions stably. Therefore, the period in which canalicular efflux can be stably observed was examined. Canalicular efflux was evaluated using FDA and tauro-nor-THCA-24-DBD on days 7–16 of the sandwich culture. Similar fluorescein images were obtained with either model substrate of biliary efflux transporters during the assay period (Fig. 6). These results suggest that canalicular efflux can be stably observed for at least 9 days.

### Observation of bile canaliculi structure by transmission electron microscopy

**Figure 7:**
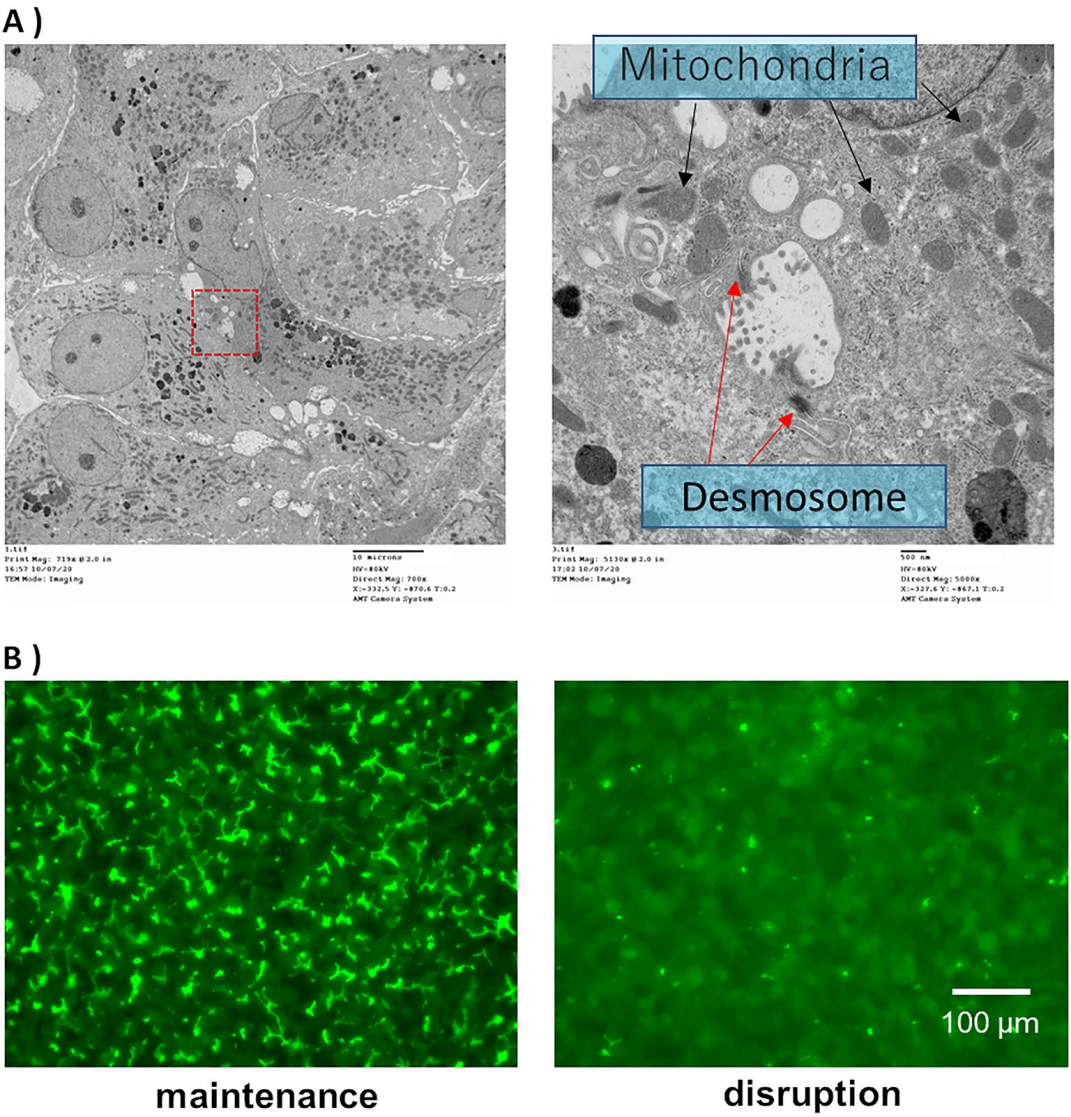
(**A**) Microstructure of bile canaliculi was observed using transmission electron microscopy. There are bile canaliculi in the dotted line in the left electron microscopy image. The right electron microscopy image is an enlarged view of the dotted line in the left that shows the microstructure of bile canaliculi. hiPSC-Heps were cultured in the LTM medium for 28 days and then sandwich-cultured in a maintenance medium for 9 days. (**B**) Accumulation of model fluorescein substrates into bile canaliculi under maintenance or disruption conditions and the microstructure of bile canaliculi. Human-induced pluripotent stem cell-derived hepatocytes (hiPSC-Heps) were cultured in a long-term maintenance (LTM) medium for 28 days and then, they were sandwich-cultured in a maintenance medium for 9 days. Fluorescence images reveal the presence of 5-(and-6)-carboxy-2′,7′-dichloro-fluorescein (CDF), which accumulated into bile canaliculi under maintenance or disruption conditions. hiPSC-Heps were cultured in the LTM medium for 28 days and then sandwich-cultured in a maintenance medium for 9 days.

Bile canaliculi maintain barrier function by virtue of their junctional complexes. The microstructure of bile canaliculi formed by the protocol constructed in this study was observed using transmission electron microscopy (TEM). TEM observations demonstrated that junctional complexes, including desmosomes, were formed adjacent to bile canaliculi (Fig. 7A).

### Effect of disruption of tight junctions on the accumulation of CDFDA in bile canaliculi

There are two different protocols for quantifying biliary efflux^21^. One protocol is to compare the amount of substrate accumulated in the cells when the substrate is taken up while maintaining or disrupting the conditions of the tight junctions. Another protocol is to compare the amount of substrate in the supernatant when the substrate is excreted under the maintaining or disrupting conditions of tight junctions after substrate uptake. Both methods require that the substrate excreted into the bile canaliculi is released into the culture supernatant by tight junction disruption in order to quantify the biliary efflux. Therefore, the effect of tight junction disruption on the accumulation of 5-(and-6)-carboxy-2′,7′-dichloro-fluorescein diacetate (CDFDA), which is an MRP2 substrate^22^, in bile canaliculi was examined. CDFDA accumulation in bile canaliculi was observed when the substrate in the cells was excreted while maintaining tight junctions (Fig. 7B). In contrast, the accumulation was not observed when the substrate in the cells was excreted by disrupting the tight junctions. This result suggests that the culture protocol for bile canaliculi formation using hiPSC-Heps is applicable for the quantitative biliary efflux evaluation.

### Expression and activity of major *CYPs* during bile canaliculi formation

**Figure 8:**
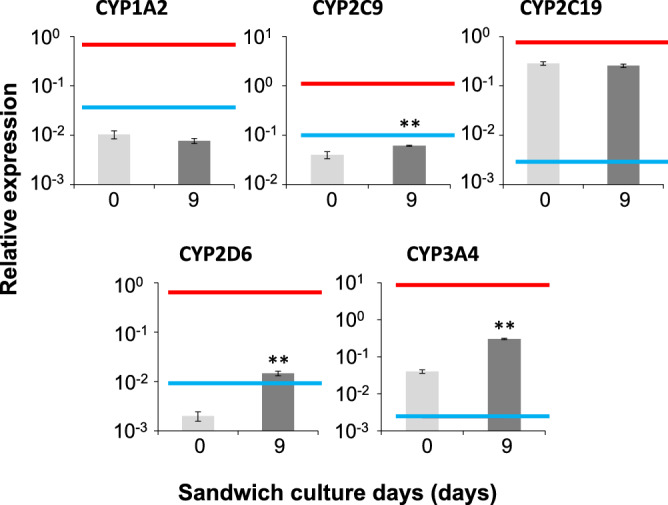
Gene expression of cytochrome P450 when the bile canaliculi were formed. Human-induced pluripotent stem cell-derived hepatocytes were cultured in a long-term maintenance medium for 28 days and then, they were sandwich-cultured in a maintenance medium for 9 days. The expression levels of major *CYPs* were measured using quantitative polymerase chain reaction before and after sandwich culture. The bar shows the relative expression levels of *CYPs*. Pooled RNA from human liver was used for the standard curve, and the expression level was set as one. The relative expression level was calculated using the equation of the line for the standard curve. The red line shows the maximum value of expression in 22 lots of human cryopreserved hepatocytes. The blue line shows the minimum value of expression in 22 lots of human cryopreserved hepatocytes. Data are presented as means ± SD. (n = 3). ** Shows that expression significantly increased compared with that before sandwich culture (P < 0.01).

Some drugs are metabolized into reactive metabolites that are associated with DILI^18^, while other drugs are metabolized into non-toxic metabolites. Therefore, drug metabolism is important for evaluating drug-induced cholestasis. The expression of major *CYPs* when the bile canaliculi formed was measured using qPCR. The expression of *CYP2D6* and *CYP3A4* increased by sandwich culture for 9 days, and that of *CYP2C19*, *CYP2D6*, and *CYP3A4* was at the same level as that in human cryopreserved hepatocytes (Fig. 8). The expression of *CYP1A2* and *CYP2C9* was slightly lower than the lowest levels in 22 lots of cryopreserved human hepatocytes. In addition, CYP2C19 and CYP3A activities almost reached the levels of human cryopreserved hepatocytes (Fig. S11).

### Scale down of culture condition for high throughput

The culture for bile canaliculi formation was originally performed in a 24-well plate, and scaling down to the 96-well plate format was attempted for the application in cholestasis toxicity tests. Extended bile canaliculi were formed when the culture was scaled down to the 96-well plate format, as was also observed in the 24-well format (Fig. S12). Additionally, similar images of the accumulation of fluorogenic substrates in bile canaliculi were found between the wells.

## Discussion

A stable formation of extended functional bile canaliculi is required to evaluate cholestasis toxicity and biliary efflux. We examined suitable culture conditions for the reproducible formation of functional bile canaliculi, aiming to apply hiPSC-Heps to cholestasis toxicity tests and biliary efflux evaluation. As a result, extended bile canaliculi could be formed with sufficient reproducibility on the whole well surface when cells were sandwich-cultured in a maintenance medium after culturing for 28 days in a long-term medium (Fig. 2). The hiPSC-Heps cultured in this protocol had transporter-dependent canalicular efflux, which is required for cholestasis toxicity tests and biliary efflux evaluation. The observation of junctional complexes adjacent to the bile canaliculi suggests that they maintain barrier function (Fig. 7). Additionally, this culture protocol was also able to form extended bile canaliculi on the whole well surface of a 96-well plate (Fig. S12). These results suggest that the proposed culture protocol using hiPSC-Heps can be applied to cholestasis toxicity tests and biliary efflux evaluation. Toxicity assays using cryopreserved human hepatocytes from the same donor cannot be continuously performed because of the limited supply of cells. In contrast, because hiPSC-Heps can be sustainably supplied from the same donor, our culture protocol may enable a stable and repeatable evaluation of cholestasis and biliary efflux toxicity. In addition, our culture protocol may be used to construct an assay model that reflects a specific disease by utilizing the patient-derived iPSC. Additionally, the bile canaliculi formed by our culture protocol maintained the functions of canalicular efflux for at least 9 days (Fig. 6). Therefore, our culture protocol may enable the evaluation of long-term cholestasis and biliary efflux toxicity. When extended bile canaliculi were formed using our culture protocol, the expression of *CYP2C19*, *CYP2D6,* and *CYP3A4* was at the same level as that in human cryopreserved hepatocytes (Fig. 8). Furthermore, we confirmed that CYP2C19 and CYP3A activities almost reached the levels of human cryopreserved hepatocytes (Fig. S11). These results suggest that cholestasis toxicity evaluation of drugs via the metabolism of these CYPs may be possible using our culture protocol.

The culture protocol for bile canaliculi formation constructed in this study is expected to be applicable to various culture vessels, such as microphysiological systems (MPS). Additionally, our culture protocol can be easily introduced at other facilities because the required culture vessels, medium, reagents, and cells are commercially available. As a future direction, we will examine the application of this culture protocol to various culture vessels, including MPS.

hiPSC-Heps were cultured for 28 days in an LTM medium before a sandwich culture to form extended bile canaliculi. The expression of *CYPs* and *ALB* increased and that of *AFP* decreased during culture in an LTM medium. These results suggested that a long-term culture in an LTM medium is effective for the maturation of hiPSC-Heps. When hiPSC-Heps were sandwich-cultured in the LTM medium instead of the maintenance medium, extended bile canaliculi were observed on the whole well surface with a phase-contrast microscope (Fig. S2B), but the accumulation of FDA in the bile canaliculi was not observed on a part of the well surface (Fig. S3). In contrast, when hiPSC-Heps were sandwich-cultured in the maintenance medium, the extended bile canaliculi were more densely observed than cells cultured in the LTM medium (Fig. S2B) and the accumulation of FDA in bile canaliculi was observed on the whole well surface (Fig. 2). Furthermore, the expression of the biliary efflux transporters, *MRP2* and *BSEP*, and hepatic uptake transporters, *OATP1B1* and *NTCP*, was increased by using the maintenance medium (Fig. 4A, Fig. S8, Fig. S13). These results suggested that the maintenance medium is effective in improving canaliculi formation and biliary efflux and may enable a more sensitive biliary efflux evaluation. AMPK signaling has been previously reported to be involved in bile canaliculi network formation^23^. Adherens junctions, which are important for maintaining the barrier function of bile canaliculi, are activated by OSM in a K-Ras-dependent manner^24^. We intend to work on studying and elucidating the mechanisms underlying the bile canaliculi formation in this culture protocol.

In this study, we were able to construct a culture protocol for the formation of extended functional bile canaliculi. Because the culture protocol constructed in this study can reproducibly and sufficiently form functional bile canaliculi, it may contribute to the creation of a highly predictable and robust cell-based assay system. We think that a future task is to study the application of this culture protocol to the evaluation of cholestasis toxicity. For quantitative evaluation of bile efflux, the substrate excreted via the biliary efflux transporter is required to be released into the culture supernatant when tight junctions are disrupted^21^. In the biliary efflux assay we performed, the accumulation of a fluorescent model substrate in the bile canaliculi was not observed when tight junctions were disrupted (Fig. 7). This result suggests that the fluorescent model substrate, which should have accumulated in bile canaliculi, can be released into the supernatant by disrupting tight junctions. Therefore, we attempted to evaluate canaliculi efflux and basolateral efflux of the fluorescent model substrate quantitatively according to the D-PREX kit protocol^25^. The ratio of canaliculi efflux and basolateral efflux could be calculated (data not shown). We plan to quantitatively evaluate the biliary efflux of a drug using LC–MS/MS in a future study. Horie et al. evaluated the bile acid-dependent toxicity caused by compounds through exposure to 26 test compounds chosen from an array of hepatotoxic drugs in the presence or absence of a bile acid mixture^26^. In future studies, we will evaluate the cholestasis toxicity of drugs in the culture protocol constructed in this study by applying their evaluation method. Currently, evaluation of the cholestasis toxicity of drugs in vitro is mainly performed using gene expression analysis of bile efflux transporters^27–30^ and by the activity measurement of bile efflux transporters in vesicles^13,14^. The cholestasis toxicity evaluation of drugs via drug metabolism is impossible using these in vitro assays. However, such an evaluation will be possible by constructing a cell-based assay system that applies our culture protocol. As a result, it is expected that the predictability of drug-induced cholestasis from in vitro data will be improved. However, care should be taken when assessing drugs involved in CYP1A2 and CYP2C9 metabolism due to the low expression level of those CYPs genes. In addition, compared to 4 lots of human cryopreserved hepatocyte, *CYP2E1* expression was also low level (Fig. S14). Therefore, we think that a future task is to increase the expression of *CYP1A2*, *CYP2C9* and CYP2E1 by examining the culture conditions. Furthermore, we will measure not only the expression of Phase I metabolism enzymes but also the expression of Phase II metabolism enzymes. Through these studies, we hope to be able to identify cholestatic drugs before the preclinical stage and, consequently, reduce the number of drugs withdrawn from the market.

## Materials and methods

### Chemicals

Fluorescein diacetate (Sigma-Aldrich, MO, USA), 5-(and-6)-carboxy-2′,7′-dichloro-fluorescein diacetate (FUJIFILM, Tokyo, Japan) and N-(24-[7-(4-*N*,*N*-dimethylaminosulfonyl-2,1,3-benzoxadiazole)]amino-3α,7α,12α-trihydroxy-27-nor-5β-cholestan-26-oyl)-2′-aminoethane-sulfonate (Genomembrane Co., Ltd. Yokohama, Japan) were used as model substrates to characterize transporter-mediated biliary excretion. MK571 (Cayman Chemical, MI, USA) was used as an MRP2 and cyclosporine A (FUJIFILM) was used as an inhibitor of BSEP.

### Culture for forming bile canaliculi

iCell hepatocytes, which are a type of hiPSC-Heps, were obtained from FUJIFILM Callular Dynamics, Inc (FCDI; WI, USA) and cultured on a collagen-coated culture plate for 5 days according to the manufacturer’s protocol. Thereafter, hiPSC-Heps were cultured in the Cellartis^®^ Enhanced hiPS-HEP Long-Term Maintenance Medium (Takara, Otsu, Japan) for 2–4 weeks. During that time, the medium was changed every 2 or 3 days. Two to four weeks later, the LTM medium was replaced with a maintenance medium for the iCell hepatocytes (FCDI), and Matrigel (Corning^®^ Inc., Corning, NY, USA) was then added to the culture (0.25 mg/mL culture medium). Until the day when the assay for evaluation of biliary efflux was performed, the medium was changed every 2–3 days, and Matrigel was layered every week.

### Biliary efflux assay

The cells were incubated for 10 min in a maintenance medium supplemented with 10 μg/mL FDA. After changing to a fresh maintenance medium, the cells were incubated to excrete fluorescein for 20 or 30 min. The cells were incubated for 30 min in a maintenance medium supplemented with 40 μM tauro-nor-THCA-24-DBD. Then, after moving the cells to fresh maintenance medium, the cells were incubated for a further 30 min to allow excretion to occur. The fluorescent model substrate was imaged using BZ-9000 (KEYENCE, Osaka, Japan).

### Inhibition assay of biliary efflux

The cells were preincubated for 30 min in a maintenance medium supplemented with MK571 (50 μM). Thereafter, the cells were incubated for 10 min in a maintenance medium supplemented with FDA (10 μg/mL) and MK571 (50 μM). Later, the cells were incubated to excrete fluorescein for 30 min after changing to a maintenance medium supplemented with MK571 (50 μM). The cells were pre-incubated for 30 min in a maintenance medium supplemented with CsA (10 μM). After pre-incubation, the cells were incubated for 30 min in a maintenance medium supplemented with tauro-nor-THCA-24-DBD (40 μM) and CsA (10 μM). Later, the cells were incubated to excrete fluorescein for 30 min after changing to a maintenance medium supplemented with CsA (10 μM). The fluorescent model substrate, which accumulated in bile canaliculi, was imaged using BZ-9000. Fluorescence images were taken at the same exposure time with or without inhibitor.

### Observation of the effect of disruption of tight junctions on substrate accumulation in canaliculi

The cells were incubated for 10 min in a maintenance medium supplemented with CDFDA to maintain tight junctions. After that, the cells were incubated for 10 min in the intact or disruption solution included in the D-prex kit (Hitachi-Hightech, Tokyo, Japan) to maintain or disrupt the tight junctions. CDFDA, which accumulates in bile canaliculi, was observed using BZ-9000.

### Immunostaining

Cells were fixed in 4% paraformaldehyde for 10 min at room temperature. The fixed samples were permeabilized using 0.1% Triton X-100 in Dulbecco’s phosphate-buffered saline (PBS; Sigma-Aldrich) for 5 min and blocked using 1% bovine serum albumin (BSA; Nakalai Tesque, Kyoto, Japan) in PBS for 30 min at room temperature. The samples were incubated overnight at 4 °C with primary antibodies (anti-MRP2 [M2 III-6] [ab3373], 1:50 dilution; Abcam, Cambridge, MA, USA) or anti-BSEP (sc-74500, 1:200 dilution; Santa Cruz Biotechnology, Pleasanton, CA, USA) in 1% BSA in PBS. This was followed by incubation with Alexa Fluor Plus 488-labeled goat anti-mouse IgG (H + L), a highly cross-adsorbed secondary antibody (A32723, 1:200 dilution; Invitrogen, Carlsbad, CA, USA) for 1 h at room temperature. Actin filaments (F-actin) were visualized using Alexa Fluor™ 594 phalloidin (A12381, 1:40 dilution; Invitrogen, MA, USA). Fluorescence images of the immunostained samples were obtained using a Nikon A1 confocal laser microscope system (Nikon Instech, Tokyo, Japan).

### Observation of bile canaliculi structure by transmission electron microscopy

For TEM, the cells were pre-fixed in 2.5% glutaraldehyde, then immersed in 0.1 M PBS at 4 °C overnight. The cells were then washed in new 0.1 M PBS and post-fixed with 1% osmium tetroxide at 4 °C for 2 h. Then, the samples were dehydrated through a graded ethanol series and embedded in epoxy resin. After ultrathin sectioning (60 nm), the sections were stained with 2% uranyl acetate and 2.7% lead citrate (Reynolds) and observed using TEM (H-7600; Hitachi High-Tech Co. Tokyo, Japan) at 80 kV of acceleration voltage.

### RNA isolation

Cultured cells were washed twice with PBS, and total RNA was isolated from the cells using the RNeasy Total RNA Extraction Kit (Qiagen, Hilden, Germany) according to the manufacturer’s instructions.

### Measurement of gene expression using TaqMan real-time polymerase chain reaction (PCR)

Reverse transcription was performed using total RNA and High Capacity RNA-to-cDNA (Thermo Fisher Scientific, Waltham, MA, USA) according to the manufacturer’s instructions. Gene expression was measured using the QuantStudio 7 flex Real Time RCR System (Applied Biosystems, CA, USA), and the following primer and probe sets were used for the detection of each gene transcript: *ALB* (Hs00609411_m1), *AFP* (Hs00173490_m1), *CYP1A2* (Hs00167927_m1), *CYP2B6* (Hs04183483_g1), *CYP2C9* (Hs00426397_m1), *CYP2C19* (Hs00426380_m1), *CYP2D6* (Hs00164385_m1), *CYP2E1* (Hs00559368_m1), *CYP3A4* (Hs00430021_m1), *CYP7A1* (Hs00167982_m1), *FXR* (Hs01026590_m1), *OATP1B1* (Hs00272374_m1), *NTCP* (HS00161820_m1), *MRP2* (Hs00960489_m1), and *BSEP* (Hs00994811_m1). Gene expression levels are presented as relative values to the human liver-derived RNA levels (BioChain Institute, Inc., Newark, CA, USA). In addition, the expression level of *CYPs* in 22 lots of human cryopreserved hepatocytes shown in Table S1 was measured for comparison.

### Measurement of metabolic activity of CYP enzymes in hiPSC-Heps with LC–MS/MS

hiPSC-Heps were incubated in William’s E Medium supplemented with the Hepatocyte Maintenance Supplement Pack (CM4000; Gibco; Durham, NC, USA) containing a cocktail of CYP probe substrates (phenacetin 50 µM (for CYP1A2), diclofenac 5 µM (for CYP2C9), mephenytoin 50 µM (for CYP2C19), bufuralol 10 µM (for CYP2D6), midazolam 5 µM (for CYP3A)) at 37 °C. After 60 min incubation, the incubation media were collected and samples were kept at − 80 °C until performing LC–MS/MS analyses. The formed metabolites of CYP probe cocktail (acetaminophen, 4′-hydroxydiclofenac, 4′-hydroxy-S-mephenytoin, 1′-hydroxybufuralol and 1′-hydroxymidazolam, respectively) were quantified with the LC–MS/MS (Prominence Ultra-Fast Liquid Chromatography (Shimadzu, Kyoto, Japan)/SCIEX Triple Quad 5500 + (AB Sciex LLC, Framingham, MA)).

### Statistical analysis

Statistical analysis of the expression data was performed using a Student’s *t* test. Values of P < 0.01 were considered statistically significant.

## Supplementary Information

Supplementary Information.

## Data Availability

The datasets used and/or analyzed during the current study are available from the corresponding author on reasonable request.
