# Supplementary material for: Construction of a culture protocol for functional bile canaliculi formation to apply human iPS cell-derived hepatocytes for cholestasis evaluation
Source: Sci Rep. 2022 Sep 7;12:15192. doi: 10.1038/s41598-022-19469-x (PMC9452549; doi:10.1038/s41598-022-19469-x)
Supplement: Supplementary file 1 — Supplementary Information. [file 41598_2022_19469_MOESM1_ESM.pdf]

Supplemental figure 1

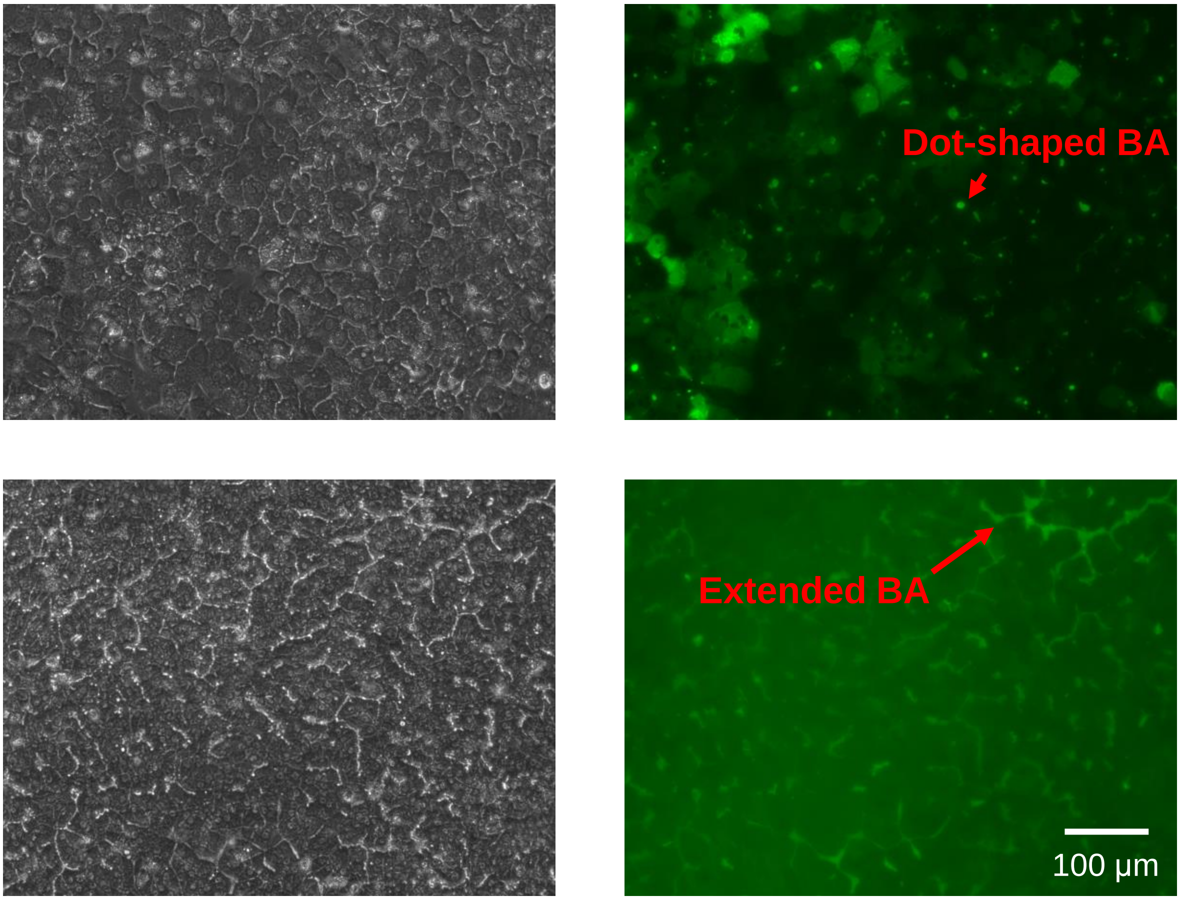

hiPSC-Heps cultured for 8 days according to the manufacturer's protocol (top). hiPSC-Heps cultured for 30 days in a long-term maintenance medium on a collagen-coated culture plate (bottom). The biliary efflux assay was performed using FDA. Phase contrast microscope image (left) and fluorescence image (right). Fluorescence images show fluorescein accumulated in bile canaliculi.

Supplemental figure 2

A)

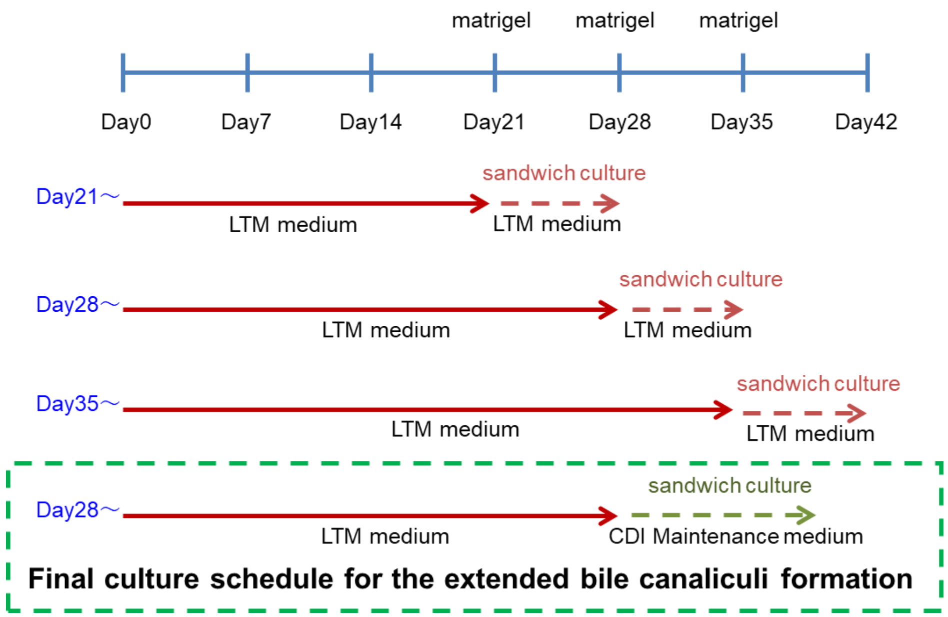

B)

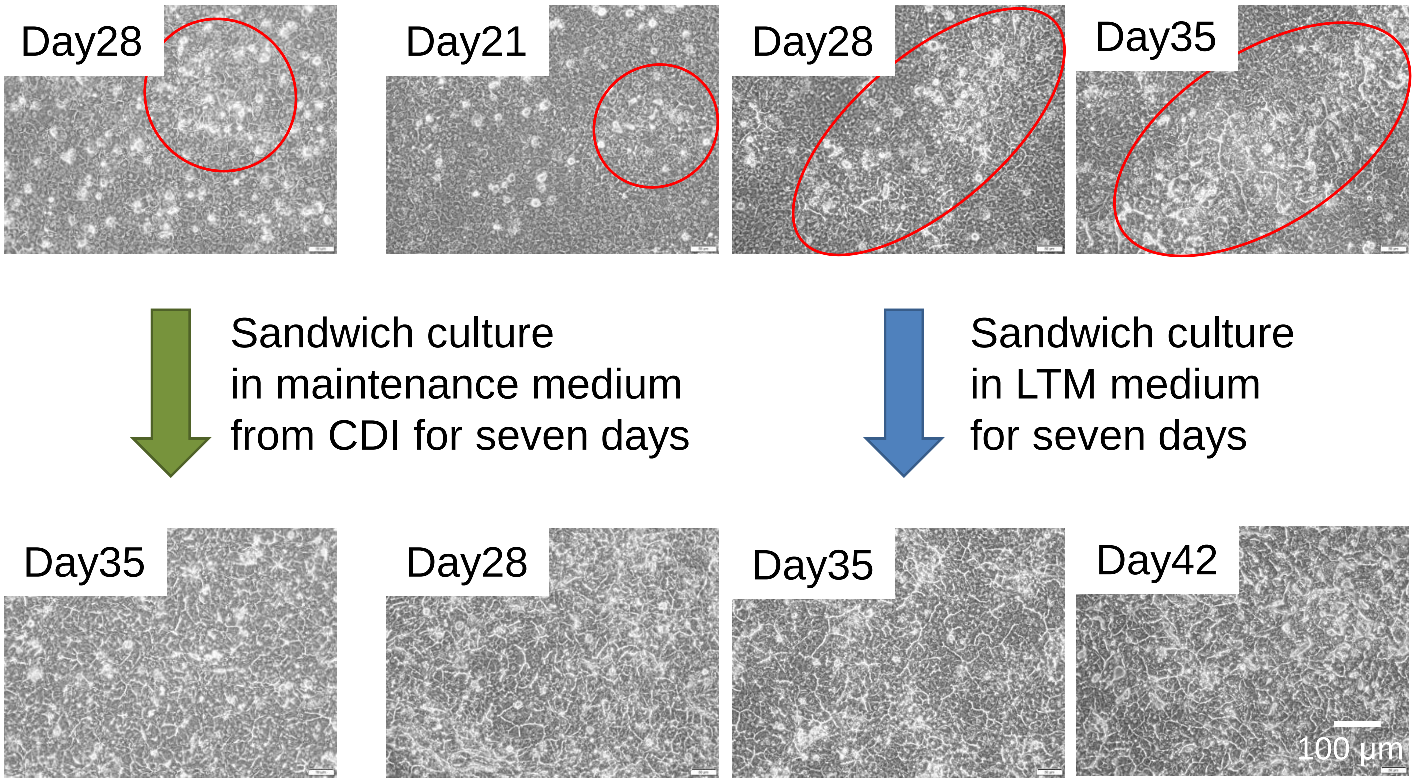

Culture schedule for the extended bile canaliculi and cell morphology before or after sandwich culture in maintenance medium (one on the left) or LTM medium (three on the right). Cell morphology was observed using a phase contrast microscope. The red circles show areas where bile canaliculi were formed before the sandwich culture.

Supplemental figure 3

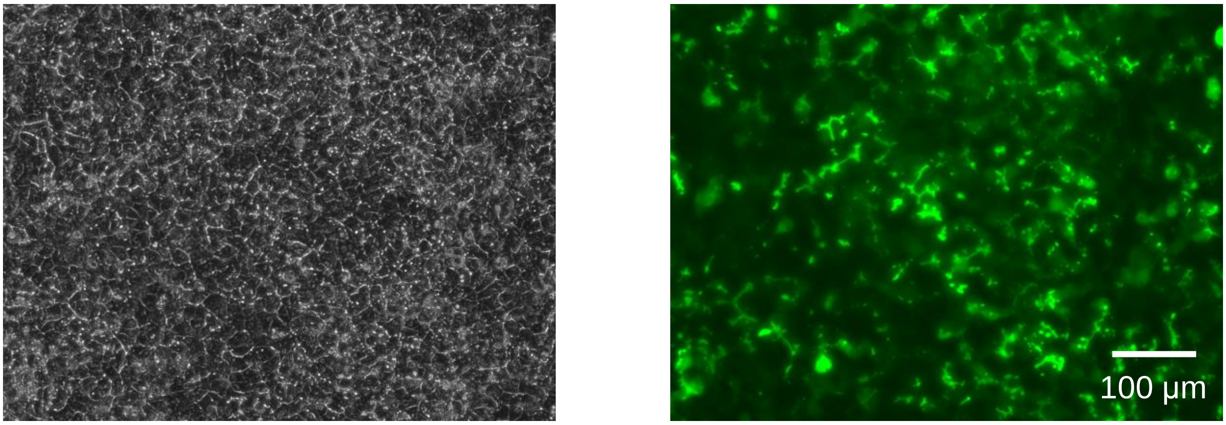

Observation of the bile canaliculi using FDA. hiPSC-Heps were cultured in a LTM medium for 28 days and then, they were sandwich-cultured in an LTM medium for 7 days. The biliary efflux assay was performed using FDA. Phase contrast microscope image (left) and fluorescence image (right) of an area where visualization of the bile canaliculi using FDA was poor. Fluorescence images show fluorescein accumulated in bile canaliculi.

Supplemental figure 4

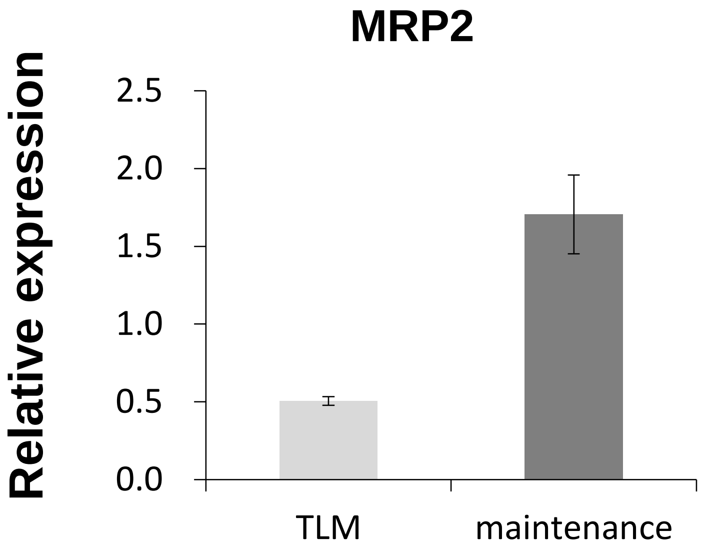

Expression of *MRP2* when hiPSC-Heps were short-term cultured in a TLM medium or maintenance medium. hiPSC-Heps were cultured in a TLM medium or maintenance medium for 4 days after a 5-day culture according to the manufacturer’s protocol. The bars show the relative expression levels of *MRP2*. Pooled RNA from human liver was used for the standard curve, and the expression level was set as one. The relative expression level was calculated using the equation of the line for the standard curve. Data are presented as means ± S.D. (n = 3).

Supplemental figure 5

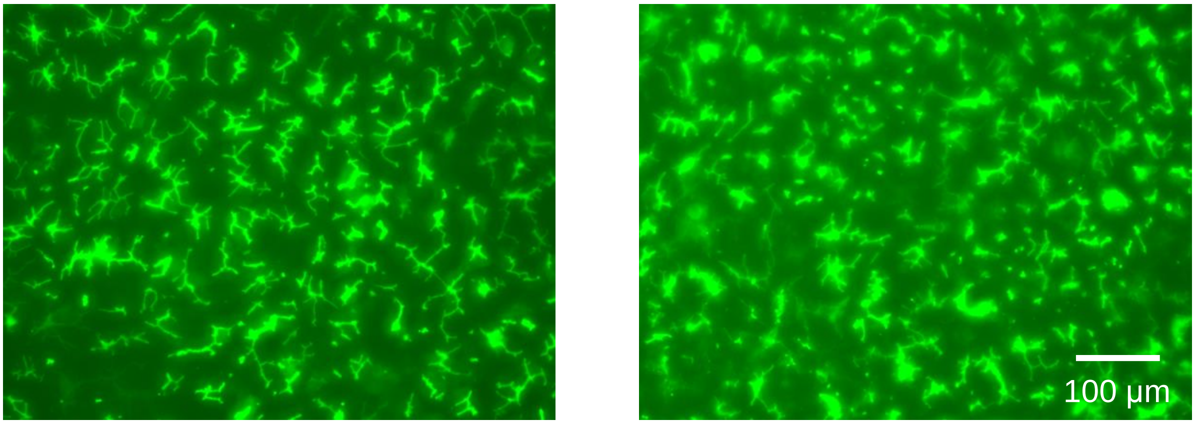

Fluorescein **Tauro-nor-THCA-24-DBD**

Observation of the bile canaliculi using FDA or tauro-nor-THCA-24-DBD. hiPSC-Heps were cultured in an LTM medium for 28 days and sandwich-cultured in a maintenance medium for another 7 days. The biliary efflux assay was performed using FDA or tauro-nor-THCA-24-DBD. Fluorescence images show that fluorescein or tauro-nor-THCA-24-DBD accumulated in bile canaliculi.

Supplemental figure 6

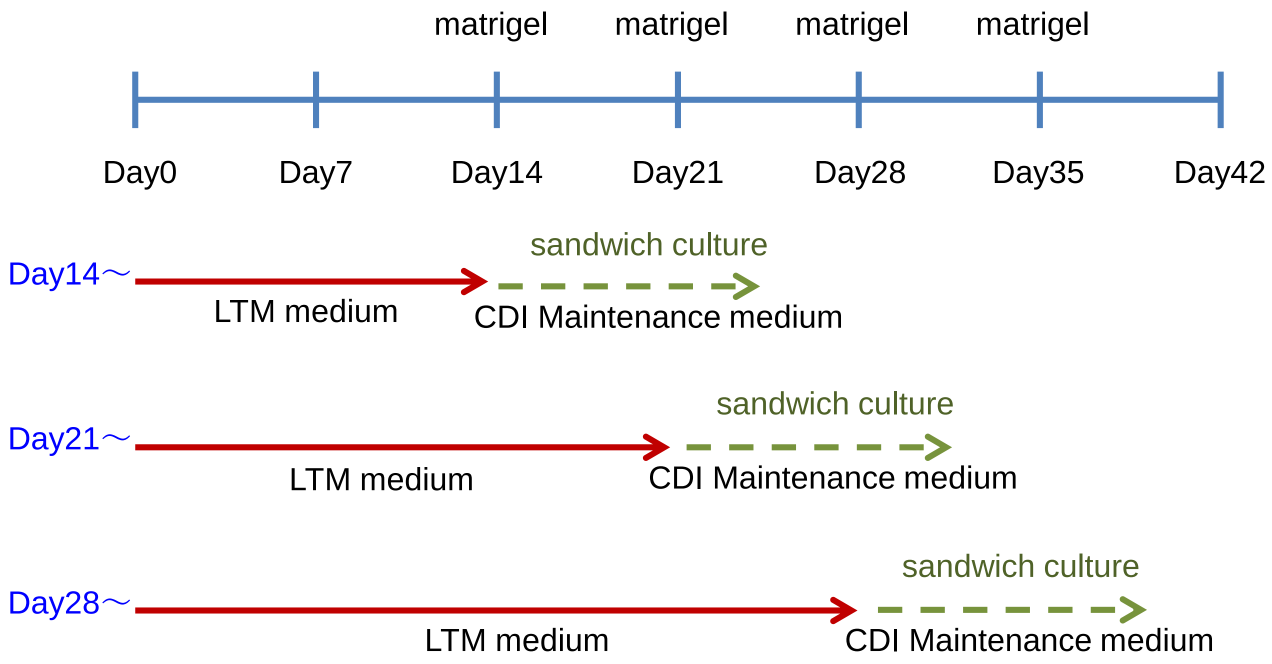

Culture schedule for the extended bile canaliculi formation using LTM medium and maintenance medium from CDI.

Supplemental figure 7

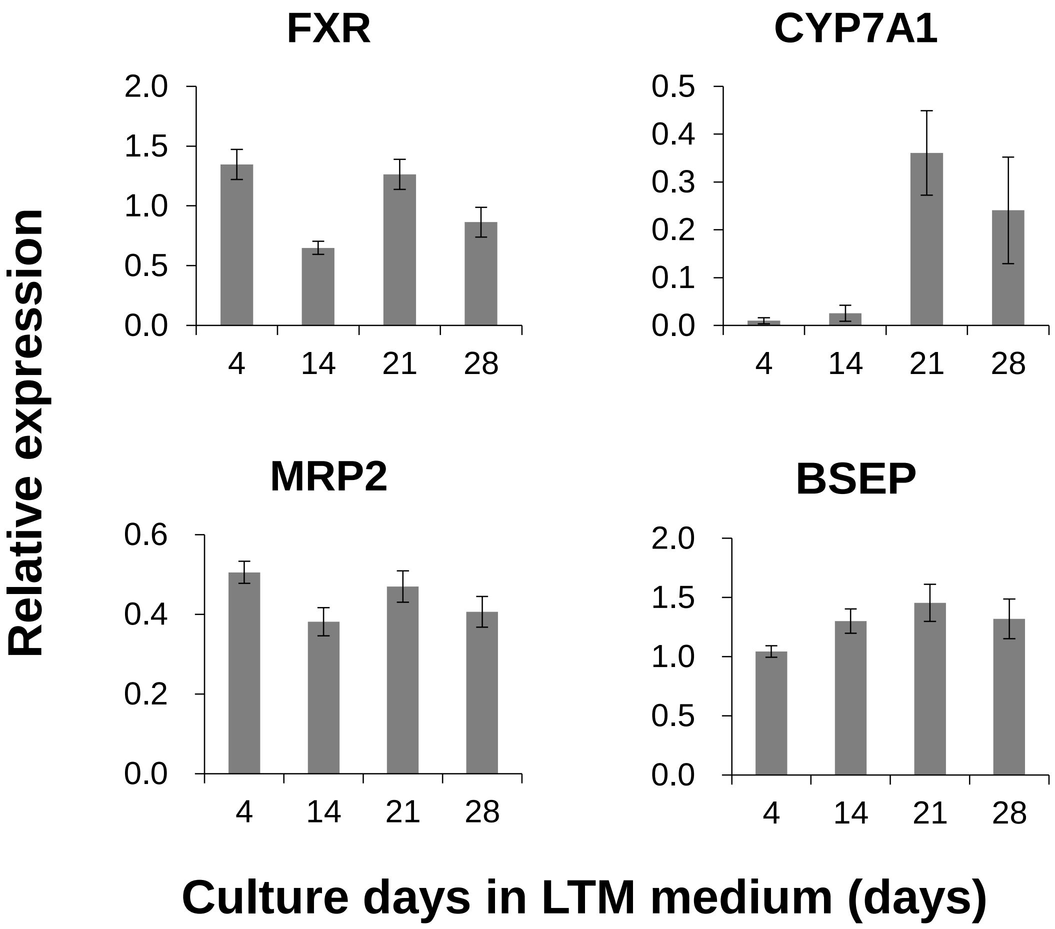

Expression of the genes related to bile acid efflux during culture in a long-term maintenance medium. The bars show the relative expression levels of *FXR*, *CYP7A1*, *MRP2*, and *BSEP*. Pooled human liver RNA was used for the standard curve, and the expression level was set as one. The relative expression level was calculated using the equation of the line for the standard curve. Data are presented as means ± S.D. (n = 3).

Supplemental figure 8

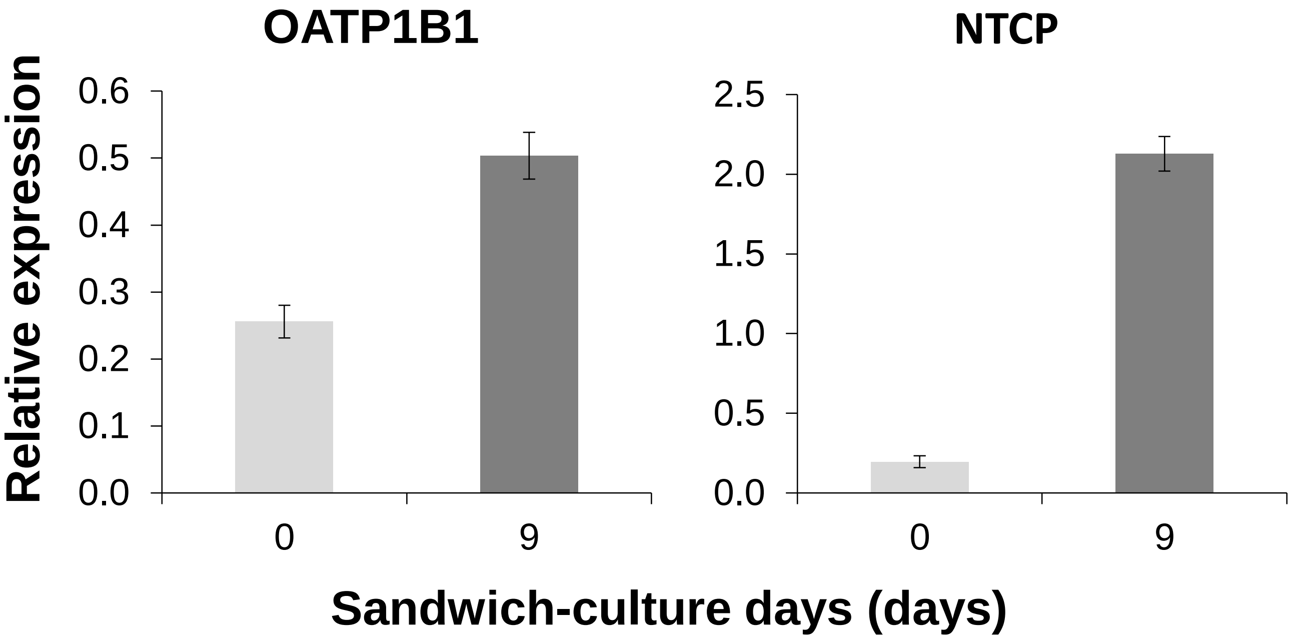

Gene expressions of *OATP1B1*, and *NTCP* when the bile canaliculi were formed. Human-induced pluripotent stem cell-derived hepatocytes were cultured in a long-term maintenance medium for 28 days and then sandwich-cultured in a maintenance medium for 9 days. The expression levels of *OATP1B1*, and *NTCP* were measured using quantitative polymerase chain reaction before and after sandwich culture. The bars show the relative expression levels *OATP1B1*, or *NTCP*. Pooled RNA from human liver was used for the standard curve, and the expression level was set as one. The relative expression level was calculated using the equation of the line for the standard curve. Data are presented as means ± S.D. (n = 3).

Supplemental figure 9

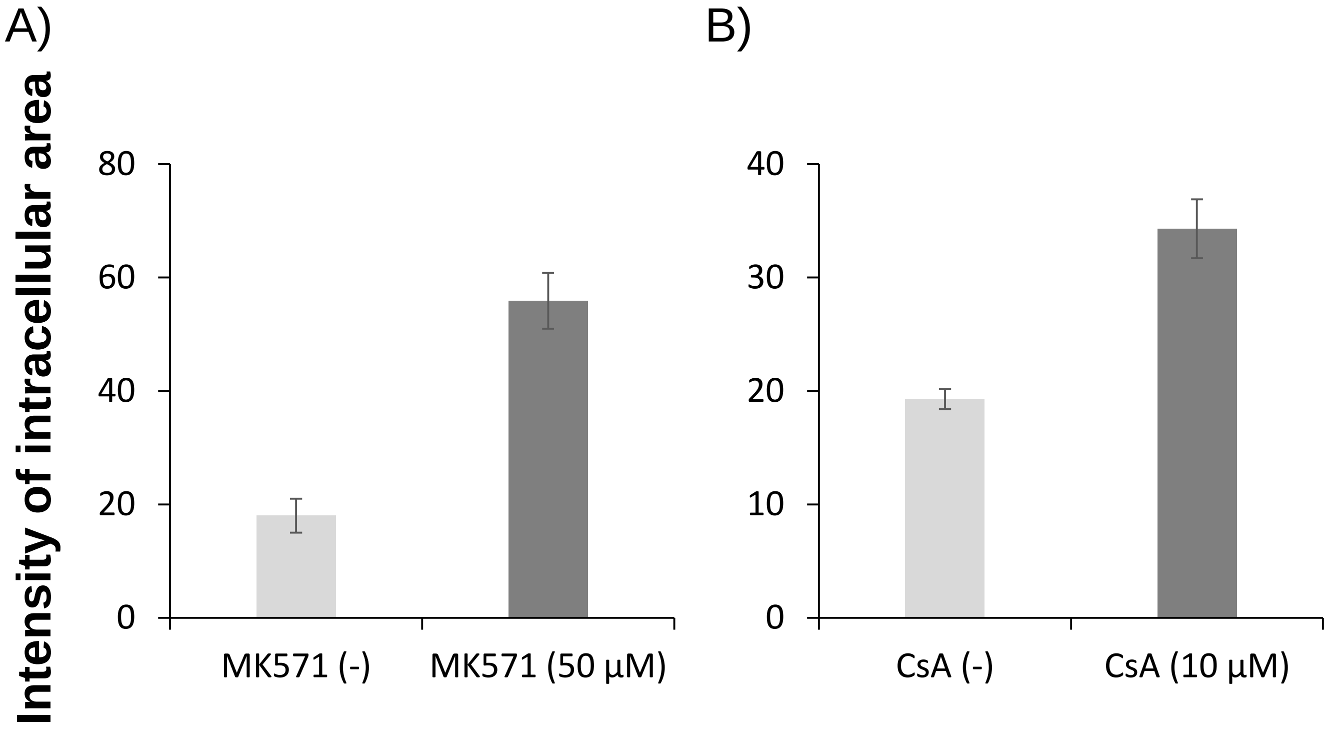

Effect of a biliary efflux transporter inhibitor on the excretion of the model substrate into bile canaliculi. Human-induced pluripotent stem cell-derived hepatocytes were cultured in a long-term maintenance medium for 28 days and then sandwich-cultured in a maintenance medium for 9 days. After that, the effect of a biliary efflux transporter inhibitor on the excretion of the model substrate into bile canaliculi was examined. Fluorescence images were taken at the same exposure time with and without inhibitor. The intensity of ten intracellular regions in the fluorescence images was measured using ImageJ (Abramoff, M., Magalhaes, P. & Ram, S. *Biophotonics Int.* **11**, 36–42 (2004)), and the average value was calculated. A) Fluorescein intensity in the intracellular area, B) tauro-nor-THCA-24-DBD intensity in the intracellular area.

Supplemental figure 10

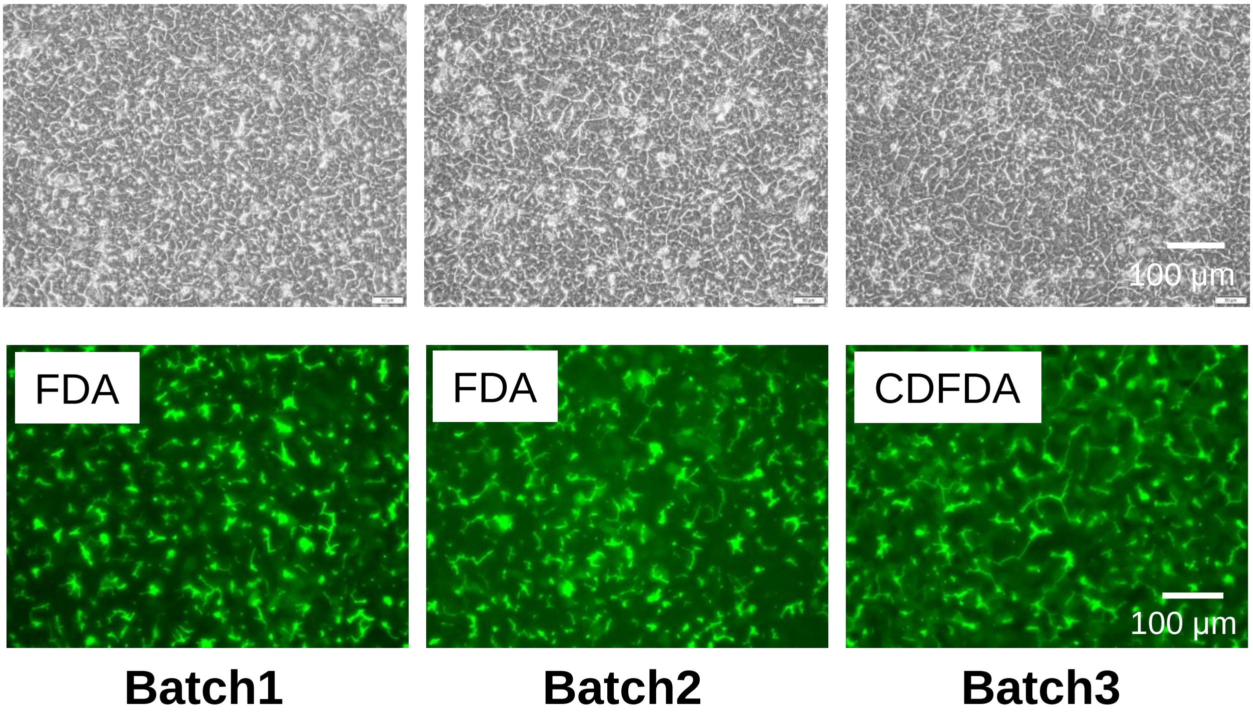

Comparison of bile canaliculi formation between batches. hiPSC-Heps were cultured in a long-term maintenance medium for 28 days and then sandwich-cultured in a maintenance medium for 9 days. Then, the biliary efflux assay was performed in each batch culture using FDA or CDFDA. Phase contrast microscope image (top) and fluorescence image (bottom). Fluorescence images show fluorescein or CDF accumulated in bile canaliculi.

Supplemental figure 11

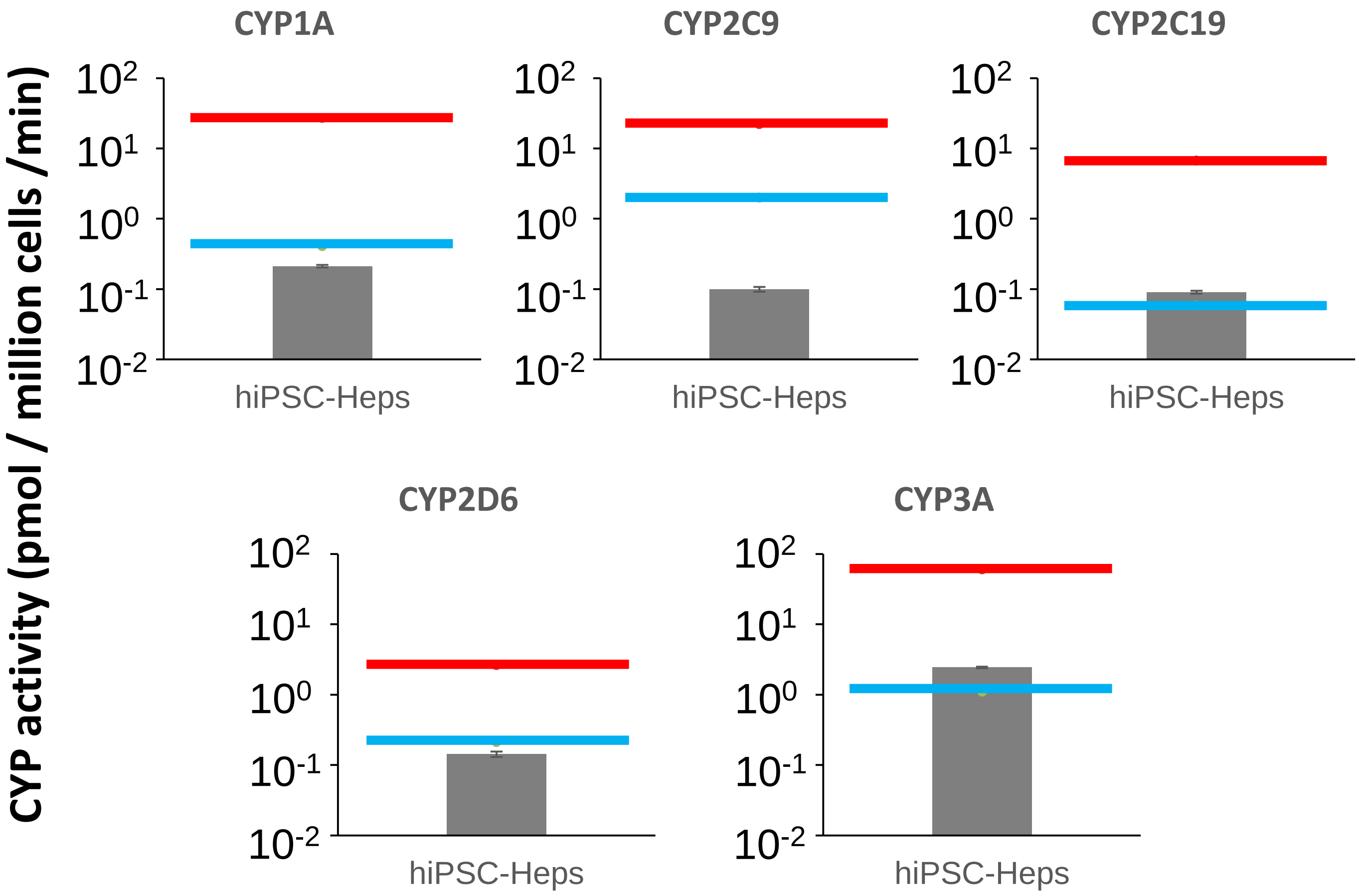

Cytochrome P450 activity when the bile canaliculi were formed. Human-induced pluripotent stem cell-derived hepatocytes were cultured in a long-term maintenance medium for 28 days and then sandwich-cultured in a maintenance medium for 9 days. Then, the metabolism test was performed. The red line shows the maximum value of activity in 8 lots of human cryopreserved hepatocytes. The blue line shows the minimum value of activity in 8 lots of human cryopreserved hepatocytes. Data are presented as means ± S.D. (n = 3).

Supplemental figure 12

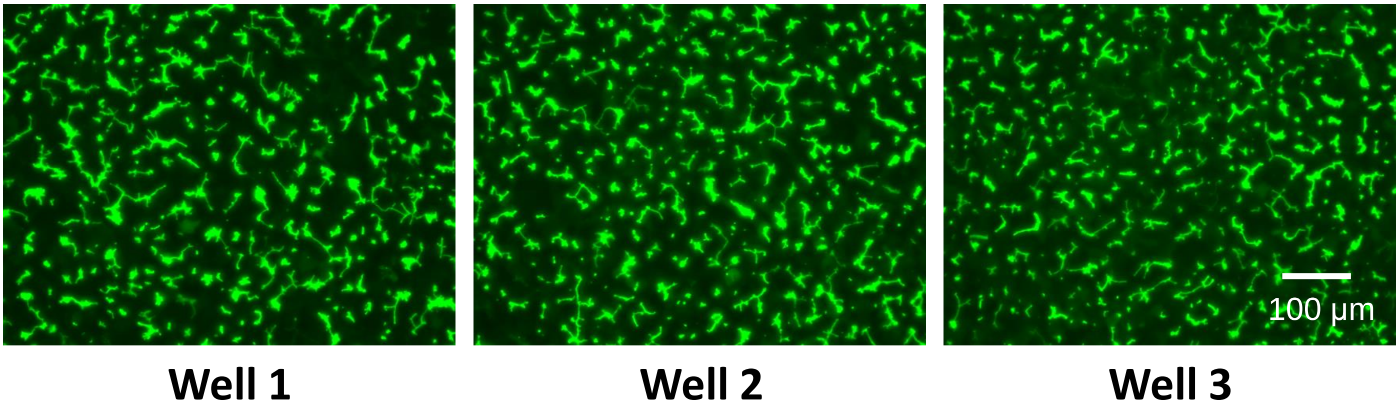

Scale-down culture for bile canaliculi formation. hiPSC-Heps were cultured in a long-term maintenance medium for 28 days and then sandwich-cultured in a maintenance medium for 10 days on a 96-well plate. Then, the biliary efflux assay was performed in three wells. Fluorescence images show CDF accumulated in bile canaliculi.

Supplemental figure 13

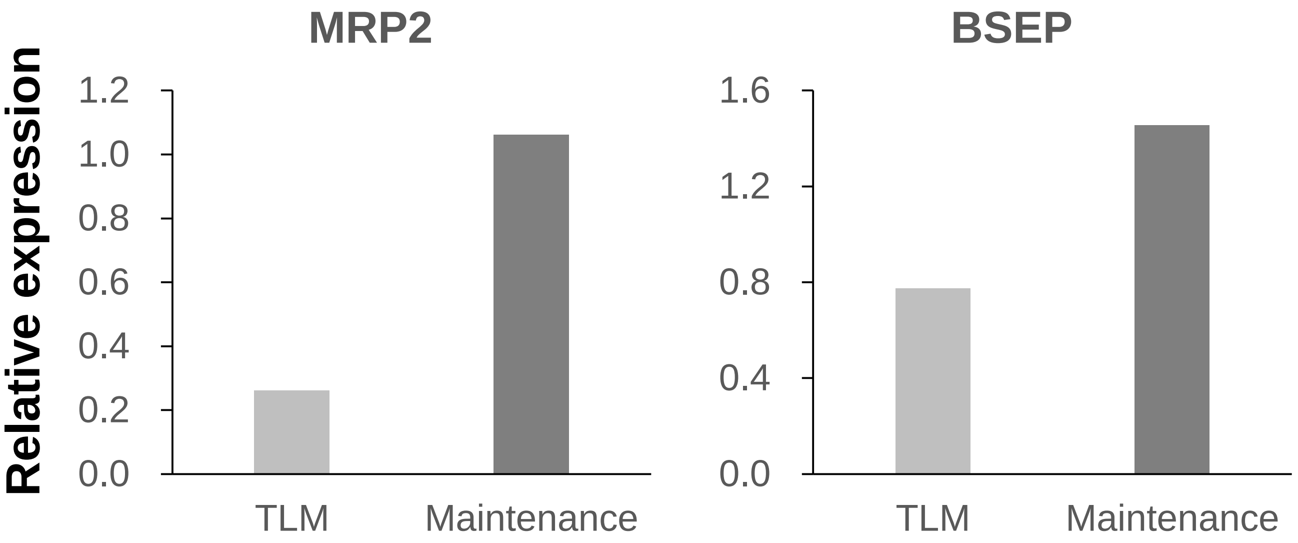

Comparison of biliary efflux transporter expression in sandwich cultures using different mediums. hiPSC-Heps were cultured in a long-term maintenance medium for 28 days and then sandwich-cultured in a long-term maintenance medium or maintenance medium for 9 days. The bars show the relative expression levels of MRP2 or BSEP after sandwich culture (n=1). Pooled RNA from human liver was used for the standard curve, and the expression level was set as one. The relative expression level was calculated using the equation of the line for the standard curve.

Supplemental figure 14

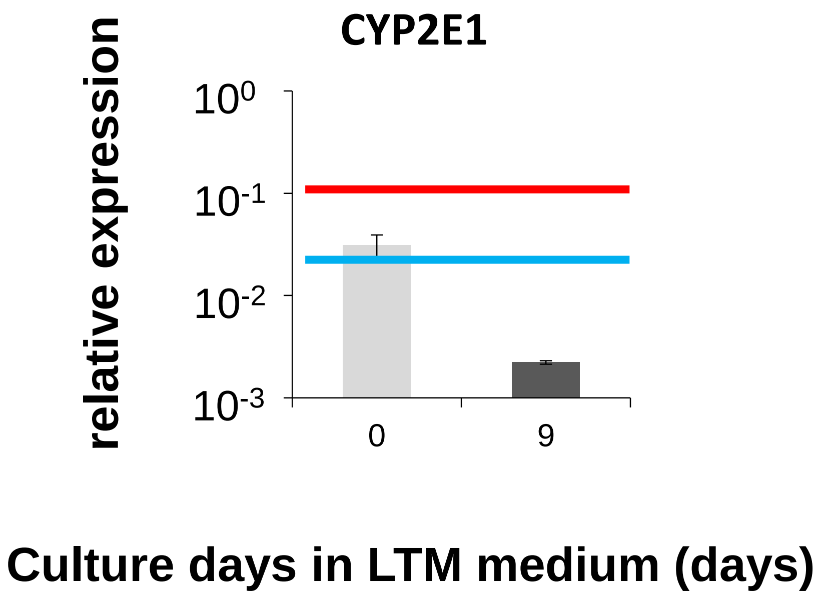

Gene expression of *CYP2E1* when the bile canaliculi were formed. Human-induced pluripotent stem cell-derived hepatocytes were cultured in a long-term maintenance medium for 28 days and then, they were sandwich-cultured in a maintenance medium for 9 days. The expression levels of major *CYPs* were measured using quantitative polymerase chain reaction before and after sandwich culture. The bar shows the relative expression levels of *CYPs*. Pooled RNA from human liver was used for the standard curve, and the expression level was set as one. The relative expression level was calculated using the equation of the line for the standard curve. The red line shows the maximum value of expression in 4 lots of human cryopreserved hepatocytes. The blue line shows the minimum value of expression in 4 lots of human cryopreserved hepatocytes. Data are presented as means ± S.D. (n = 3).

Supplemental table 1

Information on human cryopreserved hepatocytes used for comparison of CYP expressions

Vendor	Lot	Age	Gender	Race	Tobacco	Alcohol	Drug	Infectious diseases	Medical history	Cause of Death
	Lot#1	34	Male	Arabic	No	Yes	No	EBV+		Anoxia
	Lot#2	23	Male	Caucasian	Yes	Yes	Yes			Burns
	Lot#3	54	Male	Caucasian	Yes	Yes	Yes	EBV+	Joint pain, Acid reflux, Kidny stones, Seasonal allergies, Lithotripsy, Cardiac cath w/ stent, MAH ATP Neg	Cardiac
	Lot#4	28	Male	Caucasian	Yes	Yes	Yes	EBV+	Paraplegia, Kidney/bladder infections	ICH-Stroke
	Lot#5	59	Male	African American	Yes	Yes	Yes	CMV+	HTN	ICH
	(10 donor plooed)	33	Male	Caucasian	Yes	Yes	Yes	EBV+,CMV+	Kidney Stone	Drug Intoxication
		54	Male	Caucasian	Yes	Yes	Yes	EBV+	High cholesterol, Mcular degrenaration	Anoxia
		59	Male	Indian	No	Yes	No	CMV+	CAD, Sten place, HTN	Anoxia to Crdiovascular
		48	Male	Caucasian	Yes	No	No	EBV+,CMV+	HTN	Anoxia to Crdiovascular
		39	Female	Caucasian	Yes	Yes	Yes	EBV+	HTN, Lupus, Hermatoma on kidney, Pneumonia	CVA/Stroke to ICH
		51	Female	Caucasian	Yes	Yes	Yes	CMV+	Adult onset asthma, Anxiety	CVA
VendorA		42	Female	Caucasian	No	Yes	No	EBV+,CMV+	Sleep apnea, HTN, ADD, Depression, Skin cancer, Gastric bypass	Anoxia to Crdiovascular
		7	Female	African American	No	No	No		Tracheostomy, Systemic venous HTN, Cerebral HTN	CVA/ICH
		46	Female	Caucasian	No	Yes	No	CMV+	Asthma	ICH-Stroke
	Lot#6	3	Male	Caucasian	No	No	No			C-spin Injury
	(10 donor plooed)	51	Male	Caucasian	No	Yes	No			Hed Trauma/Blunt Injury
		57	Male	Caucasian	Yes	Yes	No		Alcoholic hepatitis, Aspiration pneumonia, HTN	Anoxia
		16	Male	Caucasian	No	No	No			Anoxia to Blunt Injury
		34	Male	Caucasian	Yes	Yes	No	EBV+	Borderline hypertension	CVA
		63	Male	Caucasian	Yes	Yes	No	EBV+,CMV+	HTN	Anoxia to Crdiovascular
		53	Female	African American	Yes	Yes	No	EBV+,CMV+	HTN, Laproscopic, Cholacystectomy	CVA to ICH
		53	Female	Caucasian	No	No	No	EBV+,CMV+		Anoxia
		52	Female	Caucasian	No	Yes	Yes	EBV+,CMV+	HTN	Anoxia,Asphyxiation Suicide
		34	Female	Caucasian	Yes	Yes	Yes	EBV+,CMV+	Asthma	Anoxia/Drug Intoxication
	Lot#1	44	Female	Caucasian / Hispanic	No	No	No	EBV+,CMV+		Anoxia, CVA
	Lot#2	40	Female	Caucasian	Yes	Yes	No		Asthma, epilepsy	CVA
	Lot#3	77	Female	Hispanic	No	No	No	CMV+	Asthma, HTN	CVA 2nd to ICH
VendorB	Lot#4	26	Female	Caucasian	Yes	Yes	Yes	CMV+		GSW-Head
	(10 donor plooed)	21	Female	Caucasian	Yes	Yes	Yes			Anoxia-Drug Intoxication
		40	Female	Caucasian	Yes	Yes	Yes			CVA
		55	Male	Caucasian	Yes	Yes	No			Head Trauma
		33	Female	Caucasian	Yes	No	No			Stroke
	Lot#1	64	Male	Caucasian	No	No			Hepatocellular carcinoma	
VendorC	Lot#2	26	Male	Caucasian	No	No				
	Lot#3	74	Female	Caucasian	No	No			Adenocarcinoma from a rectosigmoid cancer	
	Lot#1	56	Female	Caucasian					Duodenal neuroendocrine tumor	
VendorD	Lot#2	63	Female	Caucasian					Sigmoid adenocarcinoma	
	Lot#3	25	Female	Caucasian						
	Lot#1	54	Female	Caucasian	No	Yes	No	CMV+, EBV+		Stroke
	Lot#2	15	Male	Caucasian	No	No	No			Head Trauma
VendorE	Lot#3	45	Male	Caucasian	No	Yes	Yes			Anoxia
	Lot#4	27	Male	Caucasian	Yes	Yes	Unknown			Heroin Overdose
	Lot#5	29	Male	Caucasian	Yes	Yes	Yes			Anoxia
	Lot#6	30	Female	African American	No	No	No			Cardiac Arrest
